# Getting Research to the Policy Table: A Qualitative Study With Public Health Researchers on Engaging With Policy Makers

**DOI:** 10.5888/pcd12.140546

**Published:** 2015-04-30

**Authors:** Jennifer J. Otten, Elizabeth A. Dodson, Sheila Fleischhacker, Sameer Siddiqi, Emilee L. Quinn

**Affiliations:** Author Affiliations: Elizabeth A. Dodson, Brown School and Prevention Research Center in St Louis, Washington University in St Louis, St Louis, Missouri; Sheila Fleischhacker, The National Institutes of Health (NIH), Bethesda, Maryland; Sameer Siddiqi, Bloomberg School of Public Health, Johns Hopkins University, Baltimore, Maryland; Emilee L. Quinn, Center for Public Health Nutrition, University of Washington, Seattle, Washington. At the time of this study, Sameer Siddiqi was with the NIH, Bethesda, Maryland.

## Abstract

**Introduction:**

Little attention has been given to how researchers can best provide evidence to policy makers so that it informs policy making. The objectives of this study were to increase understanding about the current state of public health nutrition and obesity researcher practices, beliefs, barriers, and facilitators to communicating and engaging with policy makers, and to identify best practices and suggest improvements.

**Methods:**

Eighteen semistructured interviews were conducted from 2011 to 2013 with public health nutrition and obesity researchers who were highly involved in communicating research to policy makers. Interviews were transcribed verbatim, coded, and analyzed to identify common themes.

**Results:**

Study participants described wide variation in practices for communicating and engaging with policy makers and had mixed beliefs about whether and when researchers should engage. Besides a lack of formal policy communication training, barriers noted were promotion and tenure processes and a professional culture that does not value communicating and engaging with policy makers. Study participants cited facilitators to engaging with policy makers as ranging from the individual level (eg, desire to make a difference, relationships with collaborators) to the institutional level (eg, training/mentorship support, institutional recognition). Other facilitators identified were research- and funding-driven. Promising strategies suggested to improve policy engagement were more formal training, better use of intermediaries, and learning how to cultivate relationships with policy makers.

**Conclusion:**

Study findings provide insights into the challenges that will need to be overcome and the strategies that might be tried to improve communication and engagement between public health researchers and policy makers.

## Introduction

Much has been written about the importance of ensuring that research evidence is used to inform decisions such as those made in public health policy (1–3). A 2012 National Academy of Sciences report, *Using Science as Evidence in Public Policy,* states, “Science, when it has something to offer, should be at the policy table” (4). Yet the peer-reviewed public health literature has devoted little attention to understanding and improving the ways in which researchers get their work into policy pathways.

Various studies have identified many factors that hinder the translation of research evidence into public health policy, such as differences in decision making and persuasion among researchers and policy makers, ambiguous findings, and the need to balance objectivity and advocacy (5–7). A substantial literature also exists on techniques for communicating evidence-based information to policy makers; examples include developing short policy summaries and effectively framing research to resonate with policy makers (8–12). However, gaps in knowledge exist about which techniques work best when and with whom, and whether, why, and how evidence is actually used.

Important to all of the above is what researchers know and believe about engaging with policy makers and what supports them in and prevents them from effectively getting research evidence into policy pathways. However, little research exists about the current state of public health researcher practices for engaging with policy makers. What are the facilitators and barriers to policy engagement and communication? How should their work be actively communicated to policy makers? What are ways to improve the links between researchers and policy makers? The purpose of this study was to explore these questions through key informant interviews with public health researchers involved in communicating research to policy makers.

## Methods

Members of the Policy Research Impact Working Group (PRIWG) identified qualitative key informant interviews as the method best suited to begin exploring the topic (13–16). PRIWG is part of the Centers for Disease Control and Prevention–funded Nutrition and Obesity Policy Research and Evaluation Network (NOPREN, www.hsph.harvard.edu/nopren/). PRIWG exists to better understand connections between researchers and policy makers and to explore methods and best practices for researchers to make use of these connections in conducting and communicating their research.

On the basis of a literature review and PRIWG expertise, we created a semistructured interview guide that included 15 open-ended items to elicit insights from participants organized around 4 domains: 1) experience with and reasons for engaging with policy makers; 2) training, support, motivation, and barriers for communicating with policy makers; 3) assessment of what is needed to better support engaging with policy makers, including understanding how policy is formed or what constituencies want; and 4) views for improving the link between researchers and policy makers beyond the usual one-way direction of dissemination. For the purposes of the interviews, “policy makers” were defined broadly to include federal, state, or local decision makers. The interview guide was reviewed by PRIWG members who were not involved in its creation, piloted with 2 test participants, and refined for clarity on the basis of this feedback. The institutional review boards of Washington University in St Louis and University of Washington approved the study.

### Key informant interviews

PRIWG members were asked by study authors to recommend researchers who met 2 criteria: 1) their research aligned with NOPREN-relevant topics or was aimed at informing nutrition/obesity policy and 2) they were known for their leadership in working to translate and disseminate their work to policy makers. The original list included 20 participants from which 10 were recruited (November 2011–February 2012) and 4 declined. Because themes from these first 10 interviews were diverse and preliminary analysis did not show saturation of themes, a second wave of interviewees (n = 8, 2 declined) was recruited from January to November 2013 (17). These informants were drawn from the original list plus a snowball sample accrued via original informants’ interviews to optimize sample diversity.

All participants were initially contacted via email. The study purpose was explained and individuals were invited to schedule a 1-hour telephone interview. Up to 3 contact attempts were made per participant. All telephone interviews (30–60 minutes long) were conducted by lead authors (J.O., E.D.). Interviews were audio recorded and transcribed verbatim.

Two independent coders used focused qualitative data analysis techniques to systematically analyze interview transcripts (18). The use of focused coding enabled coders to analyze transcripts using the same set of thematic categories. The coders determined these categories jointly and in accordance with primary research aims. To ensure accuracy, all transcripts were coded in duplicate.

## Results

In total, 18 key informants participated in the study. Participants held primarily senior academic positions, were geographically diverse (2 Southeast, 8 Midwest, 4 Northeast, 4 West) and had expertise in public health, obesity, and nutrition. Six participants were invited but declined or did not respond to requests for participation.

The following summarizes the main themes that emerged from interviews: ways researchers communicate and engage with policy makers; factors that drive researchers to engage with policy makers; facilitators and barriers to communicating and engaging with policy makers; perspectives on and suggestions for improving the link between researchers and policy makers.

### Ways researchers communicate and engage with policy makers

Participants described a broad range of ways they communicate and engage with policy makers (Table 1), including means of information sharing and soliciting perspectives from policy makers.

**Table 1:** How Researchers Communicate and Engage With Policy Makers

Ways Researchers Communicate and Engage With Policy Makers	Description of Approach
**Direct interaction**	Either unsolicited, such as when researchers initiate legislative visits, telephone calls, emails, or texts with policy makers or their staff when relevant issues arise; or solicited, such as when researchers receive calls on specific issues, are invited to do briefings or testimony, are asked to review drafts of bills, or are asked to inform policy evaluation design
**Indirect interactions**	Included but not limited to presentations or targeted dissemination about research to federal, state, and local agencies, the media, nonprofit groups, advocacy groups, community groups, or at professional meetings/conferences where key players may be present
**Targeted dissemination products**	Creating and sending or distributing letters, peer-reviewed manuscripts, policy briefs, fact sheets, one-pagers, or bullet points to policy makers and their staff
**Professional membership groups**	Included being part of working groups that developed outputs such as policy statements; advocating for the use of practices or evidence from the field-at-large through sign-on letters, action alerts, or legislative visits
**Membership in blue ribbon groups or panels**	Often designed to inform policy at large, such as Institute of Medicine groups; transition teams; and task forces, cabinets, or roundtables formed by federal, state, or local policy makers to focus on a specific problem or task
**Planned engagement**	Included inviting policy makers to speak to academic audiences in academic settings; helping inform research development, design, or translation; writing support letters for grants of mutual topic interest; and engaging in partnerships around initiatives

### Factors driving researchers to engage with policy makers

The factors driving participants to engage with policy makers varied but generally fell into 3 categories: 1) Some stated that they were recognized experts in a policy-relevant topic, such as studying policy-affected environments like schools or daycare centers. As such, they described that they did not drive the relationship or strategic thinking about the policy implications of their research but rather that their expertise was sought by policy makers. One participant expressed, “Policy makers look for experts in topics but not experts in policy.”

2) Others shared an orientation to their work that led them to deliberately shape their research agendas to inform policy, stating that they think of their research agenda in terms of policy relevance: As one participant described, “We think of our research in terms of moving public debate. The policy world helps define the question. We consider: is it helpful in informing public opinion, in filling the knowledge gap, in the way attorneys interpret the law? You’ve got to think this way to make a difference in this world.”

3) Participants cited collaborations and relationships as the reason they became and remain involved in actively communicating with policy makers. One participant explained, “The projects . . . have really started with collaboration with people in the policy realm and sort of having them say, this is what we need. We need some evidence, we need some support.”

### Facilitators and barriers to communicating and engaging with policy makers

#### Facilitators

Participants identified several key facilitators and incentives that bolstered their policy communication efforts (Table 2). For example, they described the support for and requirements of policy engagement made by research funders, the role of institutional value placed on communicating research to policy makers, personal desire to make a difference, and opportunities for training or mentorship.

**Table 2:** Facilitators and Barriers to Policy Communication and Engagement Between Public Health Researchers and Policy Makers

Thematic Category	Participant Remarks
**Facilitators**	
Support for and requirements of policy engagement made by research funders	“The funders need to fund it, and so some of that’s happening in the obesity area.”
	“Tobacco was a reference point for NCI and NIH to fund research about policy change as opposed to only about etiology of obesity or determinants of energy balance and that kind of stuff.”
	“[A foundation] pushed me to do it [learn how to communicate with policy makers]. [The foundation] provided consultants and support for doing this and learned from folks in tobacco field.”
Support for and recognition of policy communication by academic institutions	“We actually . . . have to support junior faculty and give them credit for these kinds of relationship-building activities because it’s not an overnight process . . . it’s a 20- or 25-year process. And yet if we keep them cooped up inside working on secondary data sets the whole time and they don’t break out, they don’t get that exposure . . . they’re not going to be in a place to really make an impact later.”
Personal desire to “make a difference”	“I learned how to do it partly out of impatience. I was tired of doing research and not having it go anywhere or lead to anything.”
Training and mentorship	“How I learned it was after I was in the office and different people came in and presented their information, because they wanted some kind of legislation crafted or modified. And they were making their argument to the staff so that the senator would look at it. There was a major difference in the quality of that. And the people who came in and could sell it, and to me, they had a good database, evidence base for it and it was timely. And you could see impact, you could see why it needed to be done, why it was important. . . . Then usually those things moved more rapidly, as far as getting things out the door right away. . . . So it was the timing of things and it was how they presented it.”
	“They took me and walked me through, and had me meet people, and told me what kind of testimony worked and what kind of testimony didn’t work. And so I think that mentoring is important.”
**Barriers**	
Unsupportive academic or institutional culture	“[T]he reward system in the academy rewards the investigator for having a novel idea, and for knowledge production for its own sake much more than it rewards answering questions.”
	“[T]his is really important, I think, for junior faculty to understand . . . that the extent to which you put in your promotion packet your interest in advocacy, your interest in effectively communicating results, and more importantly, any time you spend doing it, then they’ll look and say, ‘Well, you were taking away from the time you should’ve been developing a really nationally recognized research career, getting grants or developing a teaching program.’ So not only is it not [counted?], but I think it can only be a negative within most of traditional university tenure track. So that’s why I get back to your question, which is, do you think there should be training for how to work with policy makers? I think for the traditional academic, that’s pretty far down as far as main motivators for measures of success.”
Lack of training or “know-how”	“I had no training, no mentors. I developed it over the years by doing it.”
	“Our health policy management students go, and they’ll shadow kind of the health lobbyists, and that’s great. I mean that’s the best way to learn. And I think the researchers typically don’t have that partnership, and that's why it’s hard for them.”
Perceived lack of payoff	“You can read the paper every day and see, this study says this, and it does get you a lot of buzz, and really a few meetings with policy makers gets nothing near that level of impact.”
	“For example, we just spent a few years putting together a series of papers on obesity that is supposed to speak to obesity policy. I actually think that was a much better way to spend time than to spend a lot of time with policy makers, because at the end of the day we had a day-long press conference in [city] with about 70 media outlets. We had a huge media splash . . . and then subsequently 3 different articles in the *New York Times*, and that gives you much more of an ear of policy makers than talking with policy makers and meeting with them.”
Time constraints	“Faculty are not going to have the time. They just need to know HOW to communicate. Faculty don’t have time for nitty-gritty. Faculty are really busy, especially right now in tough economic times. The reality is that there are too many other pressures. They are not going to have the time to do this.”

Abbreviations: NCI, National Cancer Institute; NIH, National Institutes of Health.

Participants cited several funders who as part of a grant application offered researchers monetary support or required them to engage with policy makers or encouraged policy communication in addition to typical dissemination through published manuscripts. These funders required researchers, when appropriate, to orient research in policy-informing ways, engage with communities, and develop well-defined dissemination plans. Some funders provided training and external experts to support these efforts.

Institutional support or culture was cited by participants as a key facilitator. Several participants stated that their institutions were discussing or developing promotion processes that would count communication and dissemination activities beyond the published manuscript and give credit for relationship-building activities.

Many participants discussed a desire to make a difference as a motivator for the policy communication work in which they engaged, noting that when policy makers make decisions, “some evidence is better than none.”

Finally, training or mentorship and work in positions outside of academia allotted a minority of participants the opportunity to learn how to engage with policy makers and why it might be beneficial or align with their personal or institutional values. However, most participants had no prior training or mentorship and most generally reported that they “learned by doing it.”

#### Barriers

Participants commented predominantly on barriers to policy communication that they observed in the field-at-large rather than those they faced personally. Barriers cited included an unsupportive culture, lack of training or “know-how,” perceived lack of payoff, and insufficient time (Table 2).

Participants consistently expressed that most research institutions do not highly value communication with policy makers and that many issues related to promotions and academic culture sustain this standard, such as promotion processes that recognize peer-reviewed publications and grants but do not take into account policy communication. Similarly, participants noted that funders often ignore this aspect of the research process.

Participants mentioned lack of training as a major barrier, indicating that because policy-related requests for research evidence often occur during times of controversy or heated decision making, researchers can feel ill-prepared and blindsided by external agendas or unfamiliar with policy-related factors that might be important in framing research evidence.

In addition, participants perceived a lack of payoff for policy work and felt that the complexity of policy making made it hard to identify or quantify the impact of their efforts. For example, participants frequently cited the media as a guaranteed way to shape and amplify one’s research messages but said that policy communication may never come to fruition, might be just a one-on-one conversation that does not produce policy change, and that the complexity of policy making may make it difficult for a policy change to directly cite a researcher’s contribution.

Finally, barriers related to time frequently emerged singularly and in connection with other themes. Participants repeatedly noted that given time limitations, they often had to choose priorities that their institutions or funders valued and that this may be a particular constraint for researchers without tenure.

### Perspectives on and suggestions for improving the link between researchers and policy makers 

#### Perspectives

Participants took mixed stances on whether and which researchers should be communicating with policy makers. Many felt strongly that all researchers should be able to articulate how their work is relevant to policy makers and be able to put it into a broader health context.

Similarly, participants expressed concern that most researchers do not understand the value of getting involved in policy work. One participant expressed, “But you have to realize: People make [policy] decisions based on no evidence, or financially invested parties drive the decisions. Isn’t some evidence better than none, even if not conclusive? I would like to see people realize the value of getting involved.”

Other participants felt that only a particular subset of applied and public health researchers should be communicating with policy makers, noting: “I don’t think everyone should be thinking this. I think there’s a group that should be motivated and well-informed and working . . . to help translate research into policy.”

Finally, some participants did not feel it was realistic for researchers to be communicating with policy makers given the system within which they operate and their differing incentive structures. This group felt that being trained how to do rigorous science was more important than figuring out what policy makers need.

My sense is that there are a lot of really smart people who think a lot about . . . moving a policy item from step A to step B to step C. So, I haven’t spent a lot of my time and energy figuring that out for any particular issue because I feel that the real added value that I can have is bringing really strong research to the table. Once that’s there, there are a lot of other people to help think through how to best understand the policy and politics process in terms of potentially unpacking that information.

#### Suggestions

Almost all participants expressed ways in which the connection between researcher and policy maker could be improved: adding training and skills, using intermediaries, and cultivating relationships. Most participants supported some form of formal training for researchers, but most were undecided about how and when it should occur. Most felt that there should be basic training about how research relates to and informs policy and that opportunities to practice were crucial. “You need to find those people and then teach them what’s known and then let them get their feet dirty in the trenches somehow.” Almost all participants indicated that systematic thinking should be done on this topic:

[I]n public health we always talk about system thinking and the importance of a systems-based approach, and yet we think about communicating with policy makers as an individual behavior and an activity that people need to . . . have some training, and just . . . if they only had better training they’d do a better job. We need to design a system . . . for success, design a system that provides rewards.

Regarding skills and elements needed to engage in productive policy communication, participants recommended that researchers

- Learn how to be effective communicators and relationship builders with policy makers.
- Know their audience. However, participant definition of this theme varied. The different ways participants described “knowing policy makers” ranged from knowing the forces that shape policy and policy makers to knowing the nitty-gritty of how policy operates and its locus-of-control and leverage points, to knowing how to frame issues in ways that are meaningful to policy makers and their constituents.
- Become a good resource or expert in some field or topic.
- Find opportunities to practice policy communication and engagement.

Participants emphasized that researchers should not be doing this work alone and should engage intermediaries (ie, groups or individuals, such as professional societies and nongovernment organizations that aim to improve the knowledge shared between networks or individuals, particularly between those who produce and use a knowledge set). Intermediaries were cited as being able to help guide researchers on policy priorities or questions that need to be answered by policy makers and the appropriate timeline for such research. In addition, intermediaries often have relationships with key policy makers and are experts at packaging information for them.

Repeatedly, participants emphasized the importance of cultivating relationships with policy makers over time. “I think the more direct that connection is, the more they [policy makers] are willing to engage, and it becomes a two-way street. The work has been most fruitful when it has been that two-way.” Although individuals can nurture these relationships on a one-to-one basis, there was a general sense that a more systematic approach to this process could be developed that included avenues for more regular interactions and opportunities for ongoing communications and to teach researchers how to enhance these relationships.

## Discussion

Policies have been cited as a useful tool for permanently and effectively changing public health behaviors — often more than many public health programs (4,6). This underscores the importance of using evidence, when available, to inform policy-making processes (4). Yet, little is known about how, why, or when researchers communicate and engage with policy makers, what is or is not working, or opportunities to improve on these practices.

This exploratory study, while addressing only one piece of the policy-making process, fills a research gap. The qualitative nature of this study provides an initial understanding about the complexity of nutrition and obesity researcher practices, beliefs, barriers, and facilitators to communicating and engaging with policy makers. Study findings provide insights into the challenges that will need to be overcome and the strategies that might be tried to improve this pathway.

Wide variation emerged in practices for communicating and engaging with policy makers along with mixed beliefs about whether and when researchers should be doing this, even among a sample of researchers who were recruited for their high levels of involvement in policy communication. This variation may reflect the absence of several related but key supports for researchers regarding policy communication: the lack of consensus on a common terminology or set of best practices or guidelines for communicating with policy makers, the lack of systematically designed training or mentorship, and the limited evidence on how research gets used in policy making (19).

Participants shared insights on possible ways to overcome barriers to policy communication with strong drivers and supports. The barriers noted occurred mostly within the academic setting (eg, a lack of formalized training and a promotion process and professional culture that does not value the practice). Nevertheless, participants cited facilitators that often overcame these barriers ranging from individual-level (eg, desire to make a difference, relationships with collaborators) to institutional-level (eg, training/mentorship support, support from the institution), and research-driven (eg, relevant topic, funder support).

Participants also agreed that the link between researchers and policy makers could be improved and suggested promising strategies such as more formal training, better use of intermediaries, and cultivating relationships. Participants in this study consistently and repeatedly underscored the need for more systematic exploration and discussion about how to guide infrastructure and training to better support researcher communication and engagement with policy makers.

To our knowledge, this is the first study to explore the current state of public health researcher practices, beliefs, barriers, and facilitators to communicating and engaging with policy makers, using qualitative interviews with researchers who are highly involved in communicating research to policy makers. This study is not without limitations. First, although diversity in geography and experience levels was sought, coverage was incomplete. Thus, the generalizability of these findings and recommendations is limited by the academic and policy research environments that were represented. Second, this exploratory study focused on researchers highly involved in communicating research to policy makers and therefore may not capture perspectives of researchers who choose not to communicate or engage with policy makers and in whom patterns of practice may differ substantially.

Future research in this area should include a synthesis of current guidelines for researchers about communicating and engaging with policy makers and to what extent these guidelines reflect our findings about what researchers are currently doing; a broader understanding of current practices in a more diverse sample; and a thorough analysis of the training that exists for researchers within and outside the research setting.
