# Erratum, Vol. 12, April 30 Release

**DOI:** 10.5888/pcd12.140546e

**Published:** 2015-05-21

In the article “Getting Research to the Policy Table: A Qualitative Study With Public Health Researchers on Engaging With Policy Makers,” a bulleted list was omitted from the Results because of a technical error. The correction was made to our website on May 1, 2015, and appears online at http://www.cdc.gov/pcd/issues/2015/14_0546.htm. We regret any confusion or inconvenience this error may have caused.

